# A liquid-liquid transition can exist in monatomic transition metals with a positive melting slope

**DOI:** 10.1038/srep35564

**Published:** 2016-10-20

**Authors:** Byeongchan Lee, Geun Woo Lee

**Affiliations:** 1 Kyung Hee University, 1732 Deogyeong-daero, Yongin, Gyeonggi 17104, Republic of Korea; 2 Korea Research Institute of Standards and Science, Daejon 34113, Republic of Korea; 3 University of Science and Technology, Daejon 34113, Republic of Korea

## Abstract

Liquid-liquid transitions under high pressure are found in many elemental materials, but the transitions are known to be associated with either *sp*-valent materials or *f*-valent rare-earth elements, in which the maximum or a negative slope in the melting line is readily suggestive of the transition. Here we find a liquid-liquid transition with a positive melting slope in transition metal Ti from structural, electronic, and thermodynamic studies using *ab-initio* molecular dynamics calculations, showing diffusion anomaly, but no density anomaly. The origin of the transition in liquid Ti is a pressure-induced increase of local structures containing very short bonds with directionality in electronic configurations. This behavior appears to be characteristic of the early transition metals. In contrast, the late transition metal liquid Ni does not show the L-L transition with pressure. This result suggests that the possibility of the L-L transition decreases from early to late transition metals as electronic structures of late transition metals barely have a Jahn-Teller effect and bond directionality. Our results generalize that a phase transition in disordered materials is found with any valence band regardless of the sign of the melting slope, but related to the symmetry of electronic structures of constituent elements.

The discovery of the first-order liquid-liquid (L-L) transition in P^1^, and low-to-high density liquid/amorphous (LDL-HDL/LDA-HDA) transition in ice^2^,^3^, Si^4^,^5^, C^6^, Ce^7^, and Ge^8^, under pressure has brought forward a common understanding that disordered phases can have a characteristic structure, like crystal phases. Since the local structure of a liquid or a glass is determined by a dominant short-range order (SRO), the phase transition in disordered phases has been explained by the change in the SRO under high pressure. In general, the characteristic geometry of SRO clusters is the result of two competing factors, i.e. maximizing a packing density and minimizing electronic bonding energy^9^: for the materials mentioned above with directional bonding (e.g. open tetrahedral order), it was possible to explain the change in the SRO when the balance between the two competing factors is perturbed by, for example, applied pressure.

Unlike covalent bonding materials with open structure of SRO giving low density, metallic glasses or liquids, from the conventional understanding, already have high packing density with icosahedral or polytetrahedral SROs at ambient pressure. This gives less chance to find a phase transformation with metallic glasses or liquids due to the SRO change by high pressure. However, surprising results against the expectation have been reported on Ce-based^10^,^11^ and Ca-based^12^ ***alloys***; even metallic glasses can transform between low- and high-density phases by pressure despite a densely close-packed structure at the ambient condition. The origin of low-to-high density amorphous (LDA-HDA) transition of the metallic glasses has been explained by volume collapse behavior with pressure, caused by the change in electronic structures^10^,^11^,^12^, meaning the competition of two factors, density and electronic bonding energy.

In the case of ***elemental transition liquid metals*** whose atoms are considered as hard spheres, it is much harder to expect low-to-high density liquid transition (LDL-HDL) than the above materials. Moreover, the LDL-HDL or LDA-HDA has been usually observed in a V-shape of melting curve in the T-P phase diagram or maximum in melting curve of *sp*-valent materials^1^,^4^,^6^ or *f*-valent rare-earth elements^7^. The V- (or negative melting slope) and maximum shapes of melting curve on Si, P, or water mean that the melting slope should change into negative or positive at certain pressure as pressure increases, implying sign change of density difference between liquid and crystal. This can be resulted from the change of liquid density, i.e. liquid-liquid transition. However, the L-L transition in elemental transition liquid metals with positive melting slope is unlikely to happen. In addition, some early transition metals (e.g., Ti and Zr), unlike water and silicon that have LDA and HDA, have been strongly suspected to have a glassy phase under high pressure, which consequently does not guarantee an observation of LDL-HDL^13^. Therefore, a negative melting slope is always a precursor to an emerging new liquid state. However, the possibility of L-L transition cannot be totally discarded in positive melting slope. Soft-core potential model proposed the L-L transition, even in positive melting curve^14^,^15^,^16^. If there is a kink on the positive melting curve, the L-L transition can be expected^17^.

In this report, we study L-L transition with Ti and Ni. These materials are chosen because they have positive melting slopes yet with opposite behaviors, mimicking soft and hard spheres for Ti and for Ni respectively, due to the difference of valence electrons. *Ab-initio* molecular dynamics calculations at 2400 K reveal the evidence of the LDL-HDL transition on liquid Ti. It is found that the local order and the electronic structure of the liquid Ti substantially change by compressing. In contrast, the local order of liquid Ni at 3000 K remains identical, confirming the nonexistence of the L-L transition in Ni. Moreover, a distinct change in velocity auto-correlation function reflects different viscosity behavior of the two liquids in the view of thermodynamics. Furthermore, the LDL-HDL transformation in liquid Ti provides a clue for the lower melting slope of the early transition metals than that of late transition metals^18^,^19^. Since the high compressibility of the liquid Ti relative to liquid Ni ultimately reduces the volume change across the solid-liquid phase boundary at high pressure and temperature, and hence, explains the lower melting slope of the early transition metals. A further study of other transition metal liquids may complete the understanding of phase transition phenomena, and may reveal unexplored high-pressure phases.

## Results

**Figure 1:**
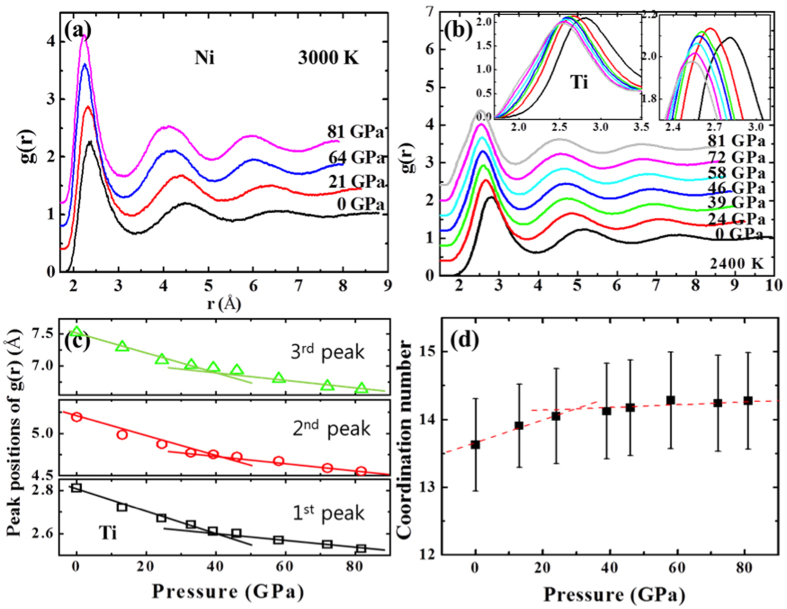
Local order changes as a function of pressure shown in, (**a**) pair correlation function (g(r)) of liquid Ni at 3000 K, and (**b**) g(r) of liquid Ti at 2400 K, (**c**) peak positions of g(r) for liquid Ti, and (**d**) the number of the nearest atoms (i.e., coordination number) in the liquid Ti with pressure. Peak broadness of the first oscillation of g(r) in the liquid Ti with pressure is appeared in the left inset in (**b**), and intensity change of the first peaks is magnified in the right inset in (**b**).

Figure 1a shows pair correlation function g(r) of liquid Ni as a function of pressure at 3000 K. The increasing intensity and narrowing width of the peaks with pressure are a typical indication of a better atomic correlation (i.e. spatial correlation) given no change of the local order of the liquid. In the case of liquid Ti, certain evidence of local order change has been revealed. That is, the intensity and width of first peak in g(r) increase and sharpen initially with pressure, but decrease and broaden after about 39 GPa (right and left inset in Fig. 1b). In addition to the transition evidenced in the slope changes in main peaks of g(r) (Fig. 1c), the number of atoms in the nearest distance (coordination number (CN)) also changes around 30~40 GPa with pressure (Fig. 1d). These changes imply that the atomic packing density of SRO in liquid Ti increases initially, and then atomic rearrangement happens nearby 30~40 GPa. The small shoulder at the short-distance side of the first oscillation in g(r) supports the configurational change within the nearest distance, signaling a liquid-liquid transition in Ti with the change of SRO.

**Figure 2:**
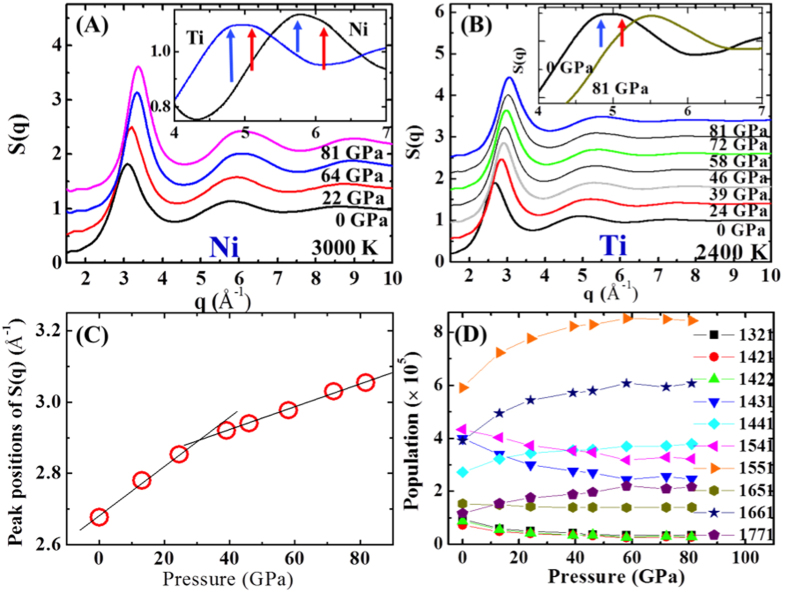
Structure factors of liquid Ni at 3000 K (**A**), and of liquid Ti at 2400 K (**B**) with pressure. Inset in (**A**) shows the second oscillation of S(q) in liquid Ti and liquid Ni at ambient pressure, showing different ‘shoulder’ intensity marked by red arrows, relative to ‘peak’ intensity marked by blue arrows. Inset in (**B**) shows the shoulder intensity in the liquid Ti decreases with pressure. The change of the first oscillation of S(q) in the liquid Ti is shown with pressure in (**C**). Honeycutt and Anderson analysis for the liquid Ti with pressure in (**D**).

Structure factors S(q) are very useful when theoretical predictions are compared with experimental measurements because S(q), but not g(r), is obtained from experiment. Moreover, we can have qualitative configurational structure information of the representative SRO from the S(q), since the S(q) is determined by the atomic distances (*r*_ij_) in the SRO, i.e., S(q) ~ sin(*kr*_ij_)/(*kr*_ij_), where *k* is wave vector, and *r*_ij_ is distance between *i*^th^ and *j*^th^ atoms. Therefore, S(q) compensates the limited information of g(r) that is averaged one-dimensional information in the real space. The changes found in S(q) are analogous to what is observed in g(r). While all the peaks of S(q) on liquid Ni do sharpen and increase with pressure, liquid Ti does not show such behavior (Fig. 2A,B). This reflects a better structural ordering in liquid Ni with pressure, but not in liquid Ti (except the initial compressing stage before the transition). Interesting feature is observed in the shape of the second oscillation of S(q) on liquid Ti which differs from that on liquid Ni (see inset of Fig. 2A). For liquid Ti at ambient pressure, the ‘peak’ (the low q side marked by blue arrows) and the ‘shoulder’ (the high q side marked by red arrows) of the second oscillation show a comparable intensity as in the insets of Fig. 2A,B. For deeply undercooled liquid Ti, the ‘shoulder’ has even slightly higher intensity than the peak^20^,^21^,^22^. Liquid Ni, to the contrary, has a distinctly different shape for the second oscillation at ambient pressure; the ‘shoulder’ intensity at high q is always lower than the ‘peak’ intensity at the low q, regardless of pressure. The shape of the second oscillation of S(q) represented by a ‘peak’-to-‘shoulder’ ratio reflects the key characteristic of the icosahedral SRO (ISRO) for liquid Ni and the defected or distorted ISRO (d-ISRO) for liquid Ti^20^,^21^,^22^,^23^.

Interestingly, the symmetry of the second oscillation of S(q) on liquid Ti becomes similar to that on liquid Ni as pressure increases (see inset of Fig. 2B). The ‘shoulder’ intensity is lower than the ‘peak’ intensity at 81 GPa, and the symmetry change of the second oscillation of S(q) on liquid Ti implies the change in SRO. In the case of liquid Ni, the symmetry of the second oscillation of S(q) remains unchanged even at high pressure, essentially identical to the case of undercooled liquid Ni^20^. In addition, the shift of the first peak position in S(q) shows different slope with pressure (Fig. 2(C)). This indicates different compressibility with pressure, since q-space is reverse to the real space.

To evaluate the configurational change, we analyze microscopic local orders using the Honeycutt and Anderson (HA) method^24^. Each index in the four-digit HA code represents the number of root atoms, common neighbor atoms, bonds among common neighbors, and different topologies sharing all first three indices in this order. For example, (1551) represents a local structure in which five neighboring atoms of a root atom are all connected like a pentagon, suggesting a characteristic of ISRO. For the HA analysis, the cutoff distance was determined with the first minimum of g(r) by fitting curves. Figure 2D shows the microstructural change in liquid Ti with pressure. As pressure increases, (1551) index indicating ISRO increases initially, while (1541) and (1431) indicating the d-ISRO decrease with pressure. This is consistent with the symmetry change of the second oscillation of S(q) with pressure from d-ISRO to ISRO. Interestingly, (1661) and (1441), which correspond to BCC or HCP ordering, also increases in fractional population, although it is relatively minor ordering. Therefore the configurational reordering occurs around 30~40 GPa which is consistent with the slope changes of main peaks in S(q) and g(r) and the CN change.

**Figure 3:**
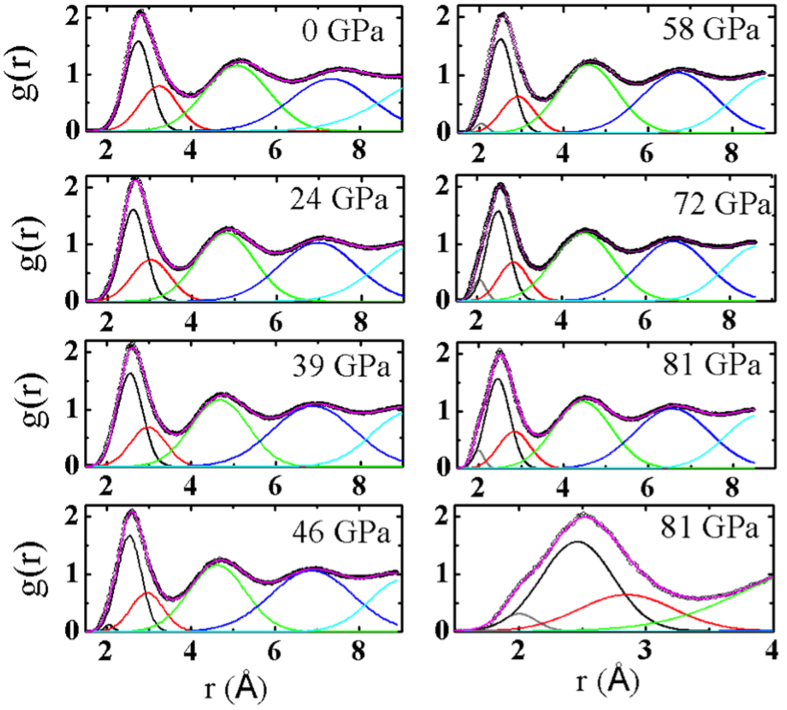
Pair correlation functions of liquid Ti with Gaussian fittings. The first oscillation of g(r) is decomposed to two Gaussian curves initially, and is fitted with three Gaussian curves from 46 GPa to 81 GPa, indicating configurational change in the nearest distance.

For more detailed understanding of the change in SRO, g(r) has been fit by Gaussian curves (Fig. 3). The first oscillation of g(r) can be fit to two Gaussian peaks at ambient pressure and 39 GPa, but to three Gaussian peaks at 46 GPa and above; the positions of the three Gaussian peaks fitting the first oscillation of g(r) at 46 GPa are located at 2.05, 2.54, and at 2.96 Å respectively. This is the origin of the broadening peak and decreasing intensity of g(r) in Fig. 1b. Above 46 GPa, the three peaks are continuously developed, causing a clear shoulder at low distance of the first oscillation of the g(r) at 81 GPa. The intensity of the first Gaussian peak at 2.05 Å increases with pressure, while that of the third Gaussian peak at 2.96 Å slightly decreases. The second Gaussian peak in the first oscillation shows relatively small increasing. Full width at half maximum (FWHM) of the peaks increases in the first peak, decreases in the third peak, and shows almost no change in the second peak. Those behaviors are consistent with almost no change of CN after 46 GPa in Fig. 2D. The change of the first oscillation of g(r) is an obvious signal for the change of configurational ordering in the nearest distance on the liquid Ti. The broadening of the first oscillation at high pressure is caused by purely electronic-structure effects as explained below, and suggestive of the necessity of first principles calculations as opposed to classical molecular dynamics.

The shortest bond of 2.05 Å at 46 GPa cannot possibly exist in the crystalline phases considering the Ti atomic size of 2.92 Å and the interatomic spacing of high-pressure phases^25^,^26^. Rather, such short bonds can be explained in connection with the results of cluster studies. For Ti clusters, d-ISRO has lower energy than ISRO by lifting degeneracy in the electronic structure^23^, and a similar electronic-structure change is observed in liquid Ti at ambient pressure^20^. The bond length can be as short as 1.92 Å for a Ti cluster due to the electronic effects and a short bond in liquid Ti is chemically more stable^23^, which leads to the conclusion that the SRO of liquid Ti as found in g(r) are likely to have short bonds, and hence, have increasingly more fragments with a stronger influence of electronic effects under pressure. In other words, the SRO change, evidenced in the population of fragmented or defected ISRO in liquid Ti at high pressure, should originate from the electronic bonding property. It is well known that the *s*-*d* electron transfer causes a phase transition in crystalline Ti^25^.

**Figure 4:**
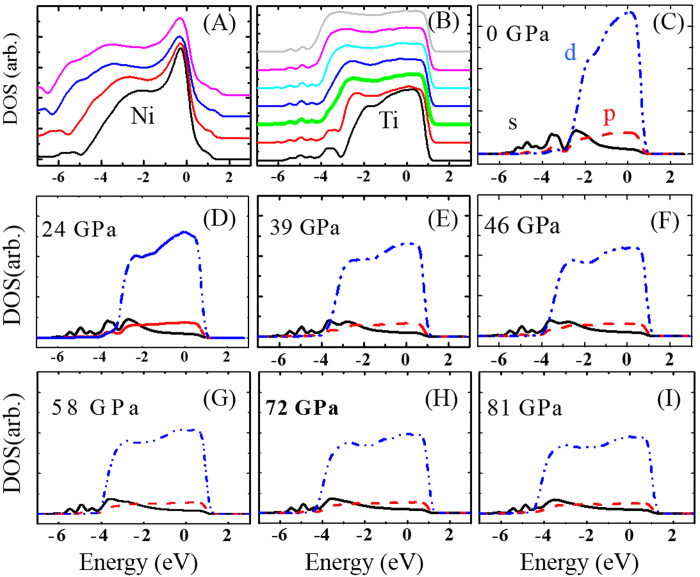
Density of states (DOS) change due to pressure. (**A**,**B**) are the total DOS of liquid Ni (0, 21, 64, and 81 GPa in order) and liquid Ti (0, 24, 39, 46, 58, 72, and 81 GPa) with pressure. The DOS at 39 GPa around the L-L transition is marked with a thick green line. (**C**) to (**J**) are the partial DOS plots with s-, p-, and d-electrons. Fermi energy is located at zero.

The increasing population of short bonds shown in g(r) is connected to the pressure-induced changes in the density of states (DOS) of liquid Ti. In Fig. 4, the clear difference in the DOS change is shown between liquid Ti and liquid Ni with pressure. While the electronic structure of liquid Ni keeps a similar shape with pressure except for the spread over a wide energy range with the reduced volume (Fig. 4A), the DOS of liquid Ti shows delocalization near −3.5 eV (Fig. 4B) along with the spread of the entire DOS. From partial DOS in Fig. 4C–I, we know that, at ambient pressure, *d*-electrons are populated around the Fermi level with a narrow bandwidth and *s*-electrons are localized in a non-bonding state at a lower energy side of the *d*-bands. As pressure increases, *d*-electrons gradually spread over a wider energy range but *s*-electrons remain stationary around −3.5 eV with intensity decreased, showing a completely different topology. The *d*-band center is shifted to a lower energy state with a broader bandwidth, and the degeneracy of *d*-electrons is lifted by a Peierls/Jahn-Teller (P/JT) distortion in which a symmetry-breaking rearrangement of atomic structures leads to higher stability and lower energy. At 39 GPa (thick green line in Fig. 4B), *s*- and *d*-bands completely overlap, suggestive of either *s*-to-*d* transition or *s-d* hybridization. Given the low intensity of *s*-electrons above the L-L transition pressure, the primary mechanism that drives the L-L transition is likely to be *s*-to-*d* transition in which the *s*-to-*d* electron ratio decreases from 0.21 at ambient pressure to 0.14 at 81 GPa. Such a charge transfer does not directly lead to the L-L transition because *s*-electrons are transferred to *d*-bands at higher energy levels below 39 GPa as evidenced in Fig. 4. Rather, the *s*-to-*d* transition increases the amount of *d*-electrons, which subsequently shows more needs of the band-center shift. Therefore, *s*-to-*d* transition has a cascading but indirect effect to the L-L transition. The change in DOS is consistent with the g(r) results. Moreover, considering that the DOS change of pressurized liquid Ti is essentially identical to that of undercooled liquid Ti, applied pressure changes SRO in the direction of breaking symmetry and/or uniformity of the atomic arrangement similar to SROs in undercooled liquid Ti^21^. As a result, the electronic binding becomes stronger and local atomic structures are stabilized. This suggests that the band energy gain is the driving force of the change in SRO.

## Discussion

**Figure 5:**
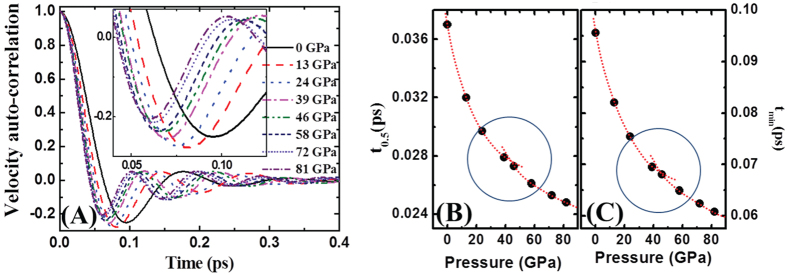
Normalized velocity auto-correlation function (VAF) of liquid Ti as a function of pressure at 2400 K (**A**). The times taking half (**B**) and minimum position (**C**) of the VAF with pressure. Both curve in (**B**,**C**) show an inflection around 40 GPa indicating the change of viscosity of the Ti liquid.

Although structural properties provide the direct evidence of a L-L transition, thermal properties, such as viscosity, also portray the phase transition as measured in experiment. The velocity auto-correlation function (VAF) is particularly useful not only because it is very sensitive to the change of local liquid density but also because it inherently reflects the viscosity change. Generally, when the liquid is compressed, the effect of pressure is two-fold, i.e. the cage effect and the viscous effect. First, an atom in the pressurized liquid metal experiences an enhanced cage effect due to interaction with surrounding neighbor atoms. This effect results in a faster oscillation with a steeper slope. Second, it also experiences an enhanced viscous effect, showing reduced magnitude in the oscillation due to a stronger interaction between atoms. The balance between the two determines the magnitude of the velocity autocorrelation in liquid metals. For liquid Ti, the magnitude of the first negative oscillation initially increases with pressure. This is consistent with the change of the first peak in g(r), i.e. increasing intensity and decreasing width of the peak. In a microscopic view, this is related to enhanced ISRO or d-ISRO formation with pressure in Fig. 2D. Because ISRO has the highest packing density, the back-scattering intensity in VAF increases: at the same time, the increased ISROs under pressure increases viscosity (or decreases diffusivity), and necessarily reduces the intensity of back-scattering^27^,^28^. Therefore the increasing-then-decreasing back-scattering intensity in VAF with pressure indicates the dominant effect switching from the cage effect to the viscous effect, which is observed around 30 ~ 40 GPa in the inset of Fig. 5A. We can also confirm this change from two distinctly different characteristic times below and above 40 GPa in Fig. 5B,C. This underlies the rearrangement of SRO in the appearance of short bonds in the first peak of g(r) around 40 GPa in Fig. 3. Consequently, the change in VAF suggests that there are two different types of Ti liquid with pressure, apparently reflecting the LDL-to-HDL transition.

Here, we discuss the type of transition in liquid Ti. Generally speaking, polymorphic transitions in liquid can show the first order transition with density anomaly, which has been observed in tetrahedrally-networked materials with covalent bonds, such as P^1^, H_2_O^2^, C^6^,^29^, and Si^5^. However, density anomaly is not a necessary condition for L-L transition. For example, in the case of a system with soft-core potential and a positive melting slope, the L-L transition occurs without density anomaly along the positive melting curve^14^,^15^. Theoretical predictions with isotropic soft-core potentials show that the region of diffusion anomaly is always larger than the region of density anomaly, and hence, a L-L transition only with diffusion anomaly can exist^30^,^31^. In the present work, we have not observed the density anomaly for the L-L transition, but the diffusion anomaly as in Fig. 5. Adam-Gibbs theory^32^ explains that diffusion can be influenced by configurational entropy (*S*_*conf*_), i.e. *D* ~ exp(−*A*/*TS*_*conf*_), where *A* is constant. Here the volume of liquid Ti shrinks with pressure initially, along with an increasing coordination number, and increasing intensity and decreasing width of the first peak in g(r). In this view, the diffusion of an atom surrounded by the nearest atoms becomes more difficult. However, if there is L-L transition causing atomic rearrangement, the atomic motion should be more relaxed during the transition. Therefore if we consider configurational change of the nearest atoms as the configurational entropy change, diffusion anomaly can be occurred, which is sensitively detected in VAF, as shown in Fig. 5. Here, entropy of liquid is composed of configurational (*S*_*conf*_) and vibrational (*S*_*vib*_) contribution, that is, *S* = *S*_*conf*_ + *S*_*vib*_. The vibrational entropy can be replaced by cage volume (*v*_*cage*_)^15^. Therefore, the following relation is obtained,


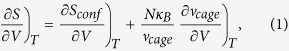


where *N* and *k*_*B*_ are Avogadro number and Boltzmann constant respectively^14^. The vibrational term in equation (1) is always positive during compression. The configurational term is positive initially with volume due to ISRO ordering shown in Fig. 2D, and then becomes negative after the L-L transition due to the rearrangement of the atoms in the nearest-neighbor distance. If the configurational contribution is small, the total entropy change with volume is positive on compression, regardless of the L-L transition. If the configurational contribution is comparable to the vibrational term, the total entropy change with volume can show a negative behavior during the transition. For the density anomaly to happen, (∂*S*/∂*V*)_*T*_ < 0. In the case of liquid Ti, the sign of (∂*S*/∂*V*)_*T*_ depends on the size of configurational contribution in equation (1). In this study, we have not observed density anomaly, i.e. (∂*S*/∂*V*)_*T*_ > 0. This may indicate a small but not negligible contribution of configurational term known from a clear change in the diffusivity or viscosity in Fig. 5. Similarly, it was reported that a positive melting curve can give L-L transition, if a kink exist on the melting curve^16^. In this case, we expect that density anomaly may or may not appear depending on the degree of the melting slope change, which should be related to the degree of configurational contribution. It should be noted that Trachenko and Brazhkin^33^ recently discussed and explained the possibility of phase transition in liquid with positive melting slope; a liquid that is placed below Frenkel line, separating solid-like oscillatory due to cage effect and gas-like diffusive components, is rigid liquid-like (or solid-like) state with well-defined short and medium range order. In a short time before particles jump to other position, the local structure is not changed in the solid-like liquid. Therefore, there is a possibility of phase transition in liquid with pressure, even in positive melting curve.

Furthermore, the L-L transition in liquid Ti may explain one of the unsolved issues in the high-pressure community; why early transition metals have lower melting slopes than late transition metals? Ross and coworkers reported that the existence of the energetically preferred local order in transition metal liquids, i.e., ISRO, lowers the melting slope^17^. They found that Al and Cu with fully filled electrons in *d*-orbital show a high melting slope while Ni and Fe with more than half filled *d*-electrons show a lower melting slope. Mo and Ta with less than half filled *d*-electrons have the lowest melting slope due to the significant P/JT distortion lowering the energy. The LDL-HDL transformation in liquid Ti provides insight into the origin of the low melting slopes. Based on Clasius-Clapeyron equation, the melting slope is given by d*T*/d*P* = ∆*V*^*l−s*^/∆*S*^*l−s*^ = *T*_*m*_ ∆*V*^*l−s*^/∆*H*_*f*_, where ∆*V*^*l−s*^ and ∆*S*^*l−s*^ are difference of volume and entropy between liquid and solid phases across the melting line, *T*_*m*_ is melting temperature, and ∆*H*_*f*_ is latent heat. In this equation, the low melting slope in early transition metals requires either small ∆*V*^*l−s*^ or large ∆*H*_*f*_ (i.e., *T*_*m*_ ∆*S*^*l−s*^). In this study, the bond shortening of liquid Ti after LDL-HDL transition results in the high-density liquid. Therefore, the liquid volume decreases at L-L transition, and gives small difference from solid volume, ∆*V*^*LDL−s*^ > ∆*V*^*HDL−s*^, lowering the melting slope. On the contrary, liquid Ni and liquid Cu^34^ did not show the local configurational order change, i.e., L-L transition, during the compression. Notice that the generation of short bonds increases the compressibility of liquid Ti compared to liquid Ni; the density of liquid Ti normalized to the ambient density is 1.35 at 39 GPa, and that of liquid Ni is 1.38 at 80 GPa, showing more volume shrink in liquid Ti than in liquid Ni. Therefore, the L-L transition in liquid Ti accounts for the lower melting slope of Ti than those of Ni and Cu, and may further implicate the similar origin for the low melting slope in other early transition metals.

Melting curves of some refractory metals are found to be lower from diamond-anvil cell (DAC) measurements than from shock wave (SW) experiments^35^. This phenomenon could be explained with the high-pressure-high-temperature polymorphism, i.e. solid-state crystalline phase transformations may appear as melts from DAC experiments. Nevertheless, it is evident that the melting curve slope is lower for Ti than for Ni at pressures due to the density increase associated with the L-L transition, which still holds considering a potential DAC/SW discrepancy in the Ti melting curve. Highly refined experiments are required to further decrease the gap between DAC and SW, and to ultimately understand the transition phenomenon entirely and accurately. The discovery in this study will trigger new DAC/SW measurements in Ti and Ni.

In summary, we have found the pressure-induced L-L transition in liquid Ti with a positive melting slope. Liquid Ti is the first system showing a new type of L-L transition theoretically predicted to have diffusion or viscosity anomaly but no density anomaly^30^,^31^ unlike L-L transitions of elemental materials with a V-shape or maxima in melting lines known so far, causing density anomaly^16^,^36^. At the atomic level, the origin of L-L transition is attributed to the *d*-band center shift due to Peierls/Jahn-Teller(P/JT) distortions, causing the rearrangement of configuration in nearest neighbor atoms. The low to high-density liquid transition reduces the volume change across the solid-liquid phase boundary, and the Clausius-Clapeyron equation predicts a lowering of the melting slope. This phenomenon provides a physical explanation to the surprisingly small melting slope (which approach zero at high pressure) observed in Ti and other transition metals at high pressure^17^,^18^, which have been under discussion for more than a decade. The predictions of an L-L transition for Ti made by computer simulations, and the recent discovery of an L-L transition in Ce^7^ by X-ray diffraction experiment, provides important evidence that polymorphic liquid structures and LDL-HDL transitions in metals are more common than had been believed. A further study on the comparison between isotropic soft-core potentials and the directional-bonding nature of early transition metals may enhance the understanding of L-L transitions.

## Methods

The equilibrium statistics were obtained from molecular dynamics using the Vienna *ab-initio* software package (VASP) with the projector augmented-wave method (PAW)^37^,^38^ and the generalized gradient approximation (GGA)^39^. The PAW potentials with 4 valence electrons (3d and 4s) and 10 valence electrons (3d and 4s) are used for Ti and Ni respectively. The system of 432 Ti or Ni atoms is equilibrated with the energy cutoff of 20 Ry at the Γ point, and thermodynamic and structural properties are obtained from the average of 5 ps after equilibration, with a 1 fs integration interval. The results of liquid Ti have been compared with those from 108 atom systems with the PAW potentials including semicore electrons (3s and 3p), but no significant difference has been observed in the transition (see Supplementary Information). The coordination number at a given pressure is calculated with the cutoff radius determined from the first minimum of g(r), and the scatter is obtained when the cutoff radius is perturbed by +/−2.5% of the cutoff. The same cutoff distance is used in the Honeycutt and Anderson analysis.

## Additional Information

**How to cite this article**: Lee, B. and Lee, G. W. A liquid-liquid transition can exist in monatomic transition metals with a positive melting slope. *Sci. Rep.* **6**, 35564; doi: 10.1038/srep35564 (2016).

## Supplementary Material

Supplementary Information
