# Supplementary material for: A liquid-liquid transition can exist in monatomic transition metals with a positive melting slope
Source: Sci Rep. 2016 Oct 20;6:35564. doi: 10.1038/srep35564 (PMC5071854; doi:10.1038/srep35564)
Supplement: Supplementary Information [file srep35564-s1.pdf]

Supplementary Material for

A liquid-liquid transition can exist in monatomic transition metals with a positive melting slope

Byeongchan Lee and Geun Woo Lee*

1. Calculation method

The equilibrium statistics were obtained from molecular dynamics using the Vienna *ab-initio* software package (VASP) with the projector augmented-wave method (PAW)[1,2] and the generalized gradient approximation (GGA).[3] The PAW potentials with 4 valence electrons (3d and 4s) and 10 valence electrons (3d and 4s) are used for Ti and Ni respectively. The system of 432 Ti or Ni atoms is equilibrated with the energy cutoff of 20 Ry at the Γ point, and thermodynamic and structural properties are obtained from the average of 5 ps after equilibration, with a 1 fs integration interval. The results of liquid Ti have been compared with those from 108 atom systems with the PAW potentials including semicore electrons (3s and 3p), but no significant difference has been observed in the transition, i.e. the decreasing intensity and peak broadening of the first peak in g(r) around the transition pressure. The primary difference is equation-of-state (EOS) prediction, and the difference between "soft" and "semicore" potentials is ~ 10 % at around 100 GPa for liquid Ti. The coordination number at a given pressure is calculated with the cutoff radius determined from the first minimum of g(r), and the scatter is obtained when the cutoff radius is perturbed by +/- 2.5% of the cutoff. The same cutoff distance is used in the Honeycutt and Anderson analysis.

2. Calculation convergence

The most critical ones pertinent to calculation accuracy at high pressure are plane-wave cutoff, valence-electron configuration of PAW potentials, and system size. We have seen that for Ti, V, and Cr at high pressure and zero temperature, a stringent setting for plane-wave cutoff and valence-electron configuration is required for accuracy in a quantitative sense, but a relaxed setting did not alter the physics of interest.[4,5] Here, a similar convergence check is repeated with the most relevant parameters.

First, the convergence against the plane-wave cutoff was checked with the cutoff energy up to 600 eV, and no significant difference has been found. Second, smaller systems, e.g. 108-atom systems, gave essentially the identical first peak of g(r). However, g(r) from a smaller system is trimmed off at a shorter distance and the analysis is restricted to short-range order. We have done extensive calculations using a semi-core potential with 108-atom systems as discussed in Section 3. Last and most important, the pseudopotential with semicore electrons, or "hard" potential (3s, 3p, 3d, and 4s), has been compared with the valence-only potential, or "soft" potential (3d and 4s only) for liquid Ti. The hard potential has the outermost cutoff

radius of 1.22 Å and the partial core radius of 1.06 Å. The soft potential has the outermost cutoff radius of 1.48 Å and the partial core radius of 1.16 Å. Both potentials have a small partial core radius such that both can be used for pressurized systems to some extent. Nevertheless the frozen core can make a difference in a few ways including core-core interaction and exchange-correlation energy; as known, the potential with the frozen core is likely to be soft. In our equation-of-state (EOS) calculations, the estimated pressure from the soft potential is around 10% lower at 100 GPa than that from the hard potential at the same volume.

The PAW formalism in principle reproduces the valence states of all-electron calculations with an enough number of plane waves inside the outermost cutoff, and from the cutoff-energy convergence test, the effect from the outermost cutoff radius is found to be negligible. In addition, the soft potential has a conservative partial core, and hence, the core and valence electrons are well orthogonalized unless the core-core overlap becomes significant. Having explicit semicore electrons could still change the results not only because the core-core interaction can potentially change the valence states but also because the exchange-correlation functional is changed. In spite of all the differences, the density of states of valence electrons from both potentials shows no significant difference under 100 GPa. Consequently, there is no reason to believe that the soft potential would give the wrong result at least in a qualitative sense around the phase transition (30 ~50 GPa). This is confirmed in g(r) and S(q).

3. Calculation results with semicore electrons

Figure S1 shows the g(r) and full width of half maximum (FWHM) of liquid Ti at 2400 K as a function of pressure. We used 108 atoms along with semicore electrons. The intensity of the first peak on pair distribution function g(r) initially increases with pressure, up to 27 GPa. This is a general behavior, since the applied pressure makes better spatial correlation between atoms in small space. However, the intensity does abruptly decrease after 27 GPa (green line), while the second peak of g(r) continuously increases with pressure. After 34 GPa, the first peak of g(r) do not increase with pressure, indicating broadening of the peak. This is an indicator of the L-L transition, since the decreasing intensity and broadening of the first peak on g(r) on pressurizing means re-arrangement of atomic configuration within the nearest distance. FWHM shows the same aspect with g(r); that is, as increasing intensity initially up to 27 GPa, the width of the peak rapidly sharpens. At transition pressure, the width slightly broadens and then shows small change with large pressure change.

Figure S2 shows the position changes of the first peak of g(r) and S(q). Both quantities clearly show the slope change near 30 GPa, which is consistent with the intensity change of the first peak in g(r) and S(q).

Therefore, we conclude that the L-L transition on liquid Ti exists using soft- and semi-core potentials, although the transition pressures are around 40 GPa and 30 GPa, respectively.

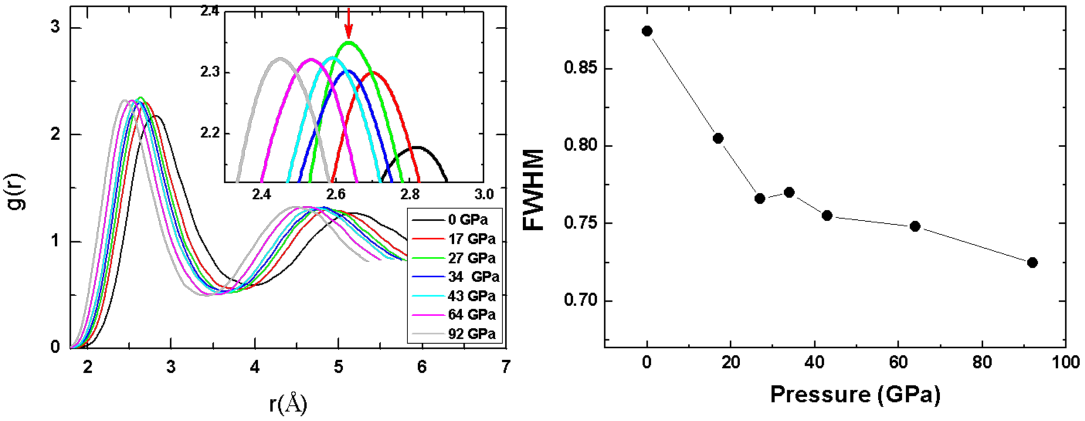

Fig. S1. Pair distribution function g(r) and FWHM of the first peak in g(r) of liquid Ti obtained by semi-core potential at 2400 K as a function of pressure.

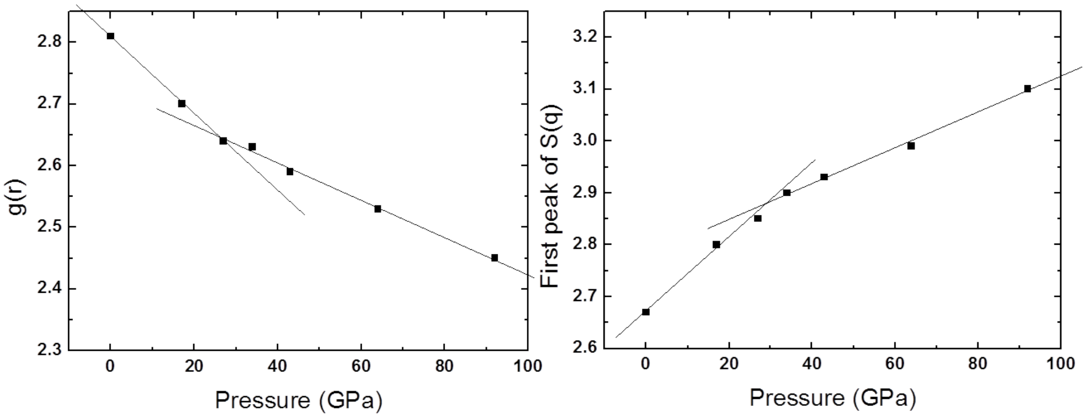

Fig. S2. Positions of the first peak on g(r) and S(q) of liquid Ti with semi-core potential

References

1. Blöchl, P. E. Projector augmented-wave method. Phys. Rev. B 50, 17953-17979 (1994).

2. Kresse, G. & Joubert, D. From ultrasoft pseudopotentials to the projector augmented-wave method. Phys. Rev. B 59, 1758-1775 (1999).

3. Perdew, J. P., Burke, K. & Ernzerhof, M. Generalized gradient approximation made simple. Phys. Rev. Lett. 77, 3865-3868 (1996).

4. Lee, B. et al. Theoretical confirmation of a high-pressure rhombohedral phase in vanadium metal. Phys. Rev. B 75, 180101(R) (2007).

5. Lee, B., Rudd, R. E. & Klepeis, J. E. Using alloying to promote the subtle rhombohedral phase transition in vanadium. J. Phys.: Condens. Matter 22 465503 (2010).
